# Efficacy of Cod-Liver Oil in Consumption

**Published:** 1854-07

**Authors:** 


### EFFICACY OF COD-LIVER OIL IN CONSUMPTION.

We find the following interesting remarks on this highly interesting subject in the last number of the *Summary of Transactions* of the College of Physicians of Philadelphia:

"Prof. Wood remarked to the College of Physicians of Philadelphia that he had looked to the obituary tables accompanying, from year to year, the reports on meteorology and epidemics, with deep interest, in reference to an important therapeutical question; the efficacy of the cod-liver oil in the treatment of pulmonary consumption. The oil has been almost universally employed in this disease; and, during the first years after its introduction, a most striking effect was observed—the number of deaths from consumption diminishing surprisingly. Now, there appeared to be no other cause to which this diminution in deaths could be attributed, excepting the use of cod-liver oil. Still, Dr. Wood had been fearful of attributing too much to the influence of this agent, inasmuch as it was known to have the effect of postponing the fatal event—of prolonging without eradicating the disease—and hence, might cause the mortality from consumption to be thrown into future years. Dr. Wood had looked with some solicitude to the report for the past year, for a solution of this question; and he was happy to find the augmentation in the deaths from consumption, in 1853, no greater than is indicated by the report. This speaks very favorably for the remedial powers of cod liver oil. There was to be anticipated an increase in the mortality from consumption during the past year, as the postponed mortality of the disease in former years would be thrown upon this. Hence, from a decrease in the proportion of deaths from consumption, since the period when it used to be between a sixth and a seventh of the whole mortality, we have a right to infer that we have gained something from the use of the oil in that disease; probably that we have cured by it one in every eight cases, with the anticipation of a still larger proportion hereafter.

